# Mandibular antegonial notch depth in postpubertal individuals: A longitudinal cohort study

**DOI:** 10.1002/cre2.577

**Published:** 2022-04-30

**Authors:** Christian Schütz, Balazs J. Denes, Stavros Kiliaridis, Gregory S. Antonarakis

**Affiliations:** ^1^ Division of Orthodontics, University Clinics of Dental Medicine University of Geneva Geneva Switzerland; ^2^ Department of Orthodontics and Dentofacial Orthopedics, Dental School/Medical Faculty University of Bern Bern Switzerland

**Keywords:** antegonial notch, growth, lateral cephalograms, mandible

## Abstract

**Objectives:**

To perform an epidemiological analysis of the antegonial notch depth in postpubertal individuals and to analyze the development of deep antegonial notches longitudinally in growing individuals.

**Material and Methods:**

Lateral cephalograms of 302 untreated 17/18‐year‐old subjects (171 males; 131 females), from the craniofacial growth legacy collection, were analysed to measure antegonial notch depth along the mandibular plane. Sex and sagittal malocclusion were investigated as possible factors influencing notch depth. In subjects with deep antegonial notches (>1.5 standard deviation) at the age of 17/18 years, earlier lateral cephalograms at 7/8 and 13/14 years were obtained, and the magnitude of notch depth analyzed longitudinally. Linear regression analyses were used to assess correlations between antegonial notch depth and other recorded variables.

**Results:**

Antegonial notch depth ranged from 0 to 5.3 mm (mean 2.0 ± 1.0 mm). Antegonial notches were significantly deeper in males (2.3 ± 1.1 mm) than females (1.5 ± 0.7 mm) (*p* < .001). Notch depth was on average 0.3 mm deeper in Class I than in Class II or III individuals (*p* = .019). Twenty‐one subjects (all male) were judged to have deep antegonial notches at the age of 17/18. In these subjects, notch depth deepened from 13/14 to 17/18 years (*p* < .001), whereas no change was observed between 7/8 and 13/14 years.

**Conclusions:**

Antegonial notch depth shows important variation in postpubertal individuals, with males having deeper notches than females on average. In those with deep antegonial notches (all males in the present sample), notch depth increases not during prepubertal growth but during the pubertal growth spurt.

## 1 INTRODUCTION

The mandible provides the site for several muscle attachments and the basal support for the mandibular dentition and the condyle, which is important for growth and temporomandibular joint (TMJ) function. The antegonial notch, located on the posterior aspect of the body of the mandible, is defined as the curvature on the lower margin of the mandible (Becker et al., 1976), which is found anterior to the gonial process.

The presence of a deep antegonial notch is often reported together with the presence of disturbed growth of the mandibular condyles both in congenital and acquired disorders (Abramowicz et al., 2014; Ali et al., 2005; Becker et al., 1976; Björk & Skieller, 1983; Brodie, 1941; Chong et al., 2008), commonly seen in the example of juvenile idiopathic arthritis (Abramowicz et al., 2014) or Treacher Collins syndrome (Chong et al., 2008). A deep antegonial notch can also be seen in those with TMJ ankylosis, with either traumatic or infectious aetiology, where a painless and chronic limitation of motion can result in an underdeveloped mandibular ramus and body on the affected side with an accompanying deep notch (Björk, 1969; Ko et al., 2005).

Björk (1963, 1969) and Björk and Skieller (1983) reported that mandibles with a backward and downward rotation during growth were associated with a pronounced antegonial notch. The antegonial notch was proposed to be used as an indicator of an individual's growth pattern (Singer et al., 1987). In the literature, some authors state that the antegonial depth may be used as an indicator of mandibular growth potential (Lambrechts et al., 1996; Singer et al., 1987), while others show no correlation between antegonial notch depth and future facial growth (Kolodziej et al., 2002). Singer et al. (1987), in a longitudinal study on treated patients, found that the antegonial notches of subjects with a deep notch tend to deepen during growth, whereas shallow notches become shallower with growth. However, these results must be interpreted with caution since it is not known if treatment can have an effect on antegonial notch development.

It is still unclear which factors play a role in determining both the depth and shape of the antegonial notch and at what time point the characteristic deep notch develops. Normative values and distributions for antegonial notch depth do not currently exist, through epidemiological studies. Moreover, it is not yet known at what age antegonial notch depth becomes substantial, and longitudinal studies are needed to investigate this. Furthermore, it would be useful to investigate whether the magnitude of the antegonial notch is linked to craniofacial morphology, in individuals with nonpathological growth.

The purpose of this study was twofold. The first aim was to identify, cross‐sectionally, in postpubertal individuals in early adulthood (17/18‐year‐old subjects) the variation in antegonial notch depth and its differences with respect to sex and sagittal malocclusion. The second aim was to longitudinally analyze the development of deep antegonial notches during normal growth to determine during which stage of growth they tend to deepen.

## 2 MATERIALS AND METHODS

The present retrospective study was based on lateral cephalometric radiographs available on the website of the American Association of Orthodontists Foundation (AAOF) craniofacial growth legacy collection (https://www.aaoflegacycollection.org) (Baumrind & Curry, 2015). Neither formal ethical approval nor supplementary informed consent was required for the present study, as all raw data (lateral cephalograms) are publicly available from the aforementioned resource. This study was carried out in accordance with the Declaration of Helsinki.

### 2.1 Epidemiology of the antegonial notch depth in postpubertal individuals

Lateral cephalograms of nonorthodontically treated subjects at the age of 17/18 years, corresponding to early adulthood, were desired. The AAOF craniofacial growth legacy collection was searched for all lateral cephalograms at the age of 17/18, provided that lateral cephalograms of these same individuals were also available at earlier ages, in order to complete the second longitudinal part of the study which is described subsequently. All available subjects were included, in order to minimize selection bias.

Three hundred and two lateral cephalograms at the age of 17/18 years were identified. Each lateral cephalogram was incorporated into OsiriX imaging software (Rosset et al., 2004). To calibrate the lateral cephalograms with regard to magnification, the instructions given by the AAOF were used. OsiriX software was used to calculate the coordinates exported using the four fiducial landmarks, which were embedded in the digital images, ensuring that any enlargement was accounted for and measurements were carried out according to true size.

**Figure 1:**
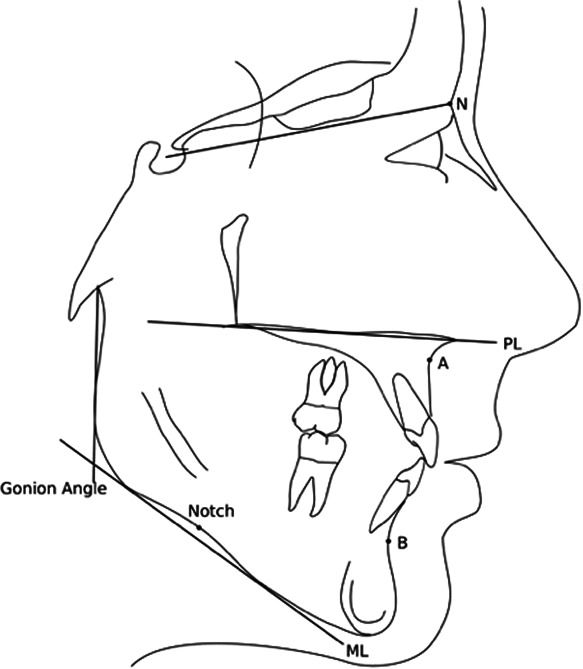
Cephalometric landmarks used were: A, A‐point; B, B‐point; Gonial angle, defined as the angle between a line going through articulare tangent to the posterior border of the ascending ramus, and ML; ML, mandibular line (defined as a line tangent to the lower border of the mandible); N, nasion; Notch, the deepest point of the concavity of the antegonial notch; PL, palatal line (defined as a line going through the anterior and posterior nasal spine).

For each subject, the depth of the antegonial notch was measured as the distance along a perpendicular line from the deepest point of notch concavity to a tangent through the two points of greatest convexity on the inferior border of the mandible, using the method of Lambrecht et al. (1996) (Figure 1). If there were double contours of the anatomical structures due to different projections of the left and right sides of the head, the mean between the two contours was used. The mean notch depth, as well as its distribution, was evaluated in the whole sample, with sagittal malocclusion (Class I, Class II, and Class III) and sex as independent variables. Sagittal malocclusion was determined based on data presented on the AAOF craniofacial growth legacy collection website.

### 2.2 Longitudinal deep antegonial notch changes

Based on the distribution of notch depth in the total sample of 302 individuals, a deep antegonial notch was defined as a notch depth corresponding to that greater than 1.5 standard deviation from the mean. This cutoff point was chosen so as to include a little more than 5% of the population, which represents the upper extreme of notch depth. The lateral cephalometric headfilms from these individuals were obtained at the age of 7/8 years (corresponding to prepubescence) and 13/14 years (corresponding to adolescence), similarly to the three approximate age categories used by Kolodziej et al. (2002).

**Figure 2:**
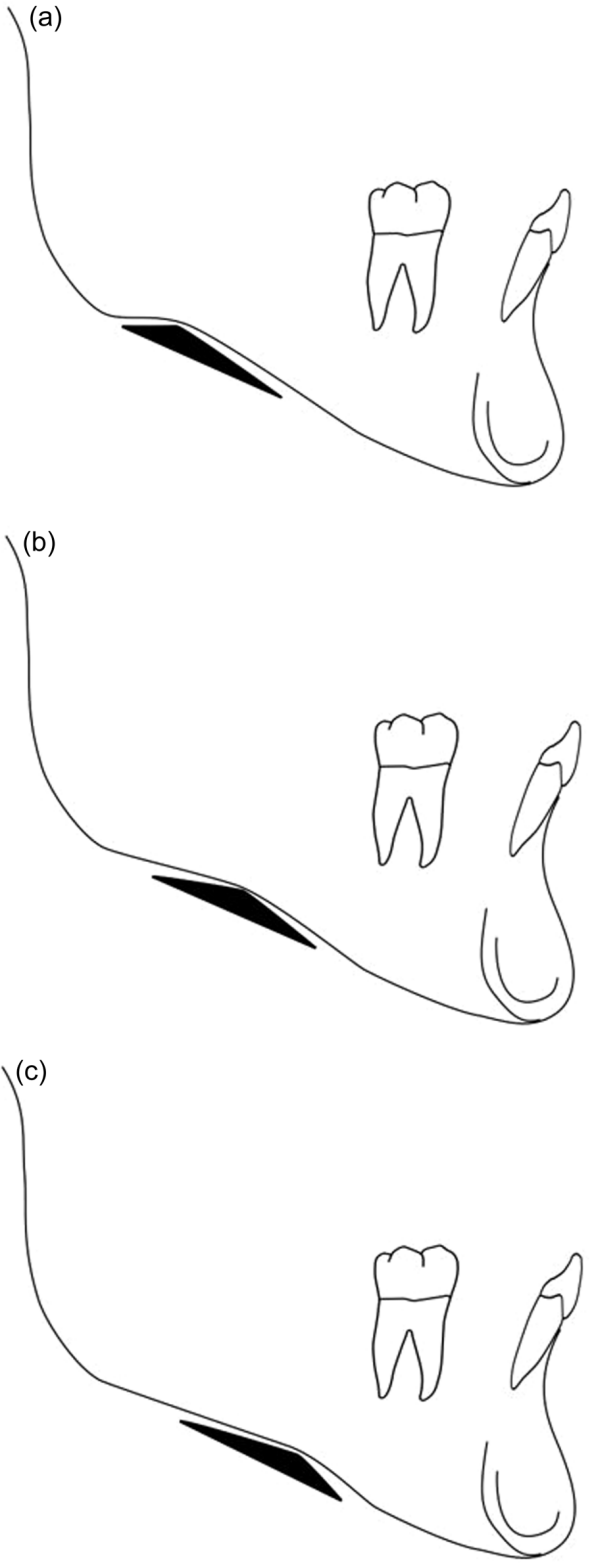
Morphology of the antegonial notch. (a) Type 1: deepest point of the notch located on the posterior aspect of the concavity. (b) Type 2: deepest point of the notch located in the middle of the concavity. (c) Type 3: deepest point of the notch located on the anterior aspect of the concavity.

The same method for antegonial notch depth measurement was carried out as previously described, on lateral cephalograms. Additional measurements were carried out defining both sagittal and vertical skeletal relationships, namely, the ANB (A point, nasion, B point) angle according to Steiner, as well as the vertical jaw relationship (intermaxillary angle: palatal plane–mandibular plane) and the gonial angle according to Bjork (Figure 1). These were measured at all three time points (7/8, 13/14, and 17/18 years) in the subjects with deep antegonial notches at the end of growth. Additionally, the antegonial notch types were defined (Types 1, 2, and 3; Figure 2), according to the study from Porwolik et al. (2015), for these subjects at the ages of 7/8 and 17/18 years. Briefly, this typology of the antegonial notch was based on the position of the deepest point of the notch within the antegonial notch concavity, with Type 1 denoting the deepest point of the notch on the posterior aspect of the concavity, Type 2 in the middle of the concavity, and Type 3 on the anterior aspect of the concavity.

### 2.3 Error of method

Using the true random number service (https://www.random.org), 10% of lateral cephalograms at 17/18 years (*n* = 30) were randomly chosen and retraced by the same operator to evaluate the error of the measurements. The systematic error was calculated using paired *t‐tests*, and random error using Dahlberg's formula (Houston, 1983).

### 2.4 Statistics

Analysis of the antegonial notch depth variation was performed by analysis of variance with independent variables such as sex and molar class and with a Tukey post hoc test. Correlations between antegonial notch depth and sagittal or skeletal cephalometric variables, as well as changes with age, were evaluated using linear regression analysis. Changes in antegonial notch typology over time were evaluated using *χ* ^2^ statistics. All analyses were performed using JASP (v.0.11.1).

## 3 RESULTS

### 3.1 Error of method

No significant systematic error was found (*p* > .05). The maximum random error was found to be 0.3 mm with respect to antegonial notch depth.

### 3.2 Epidemiology of the antegonial notch depth in postpubertal individuals

**Figure 3:**
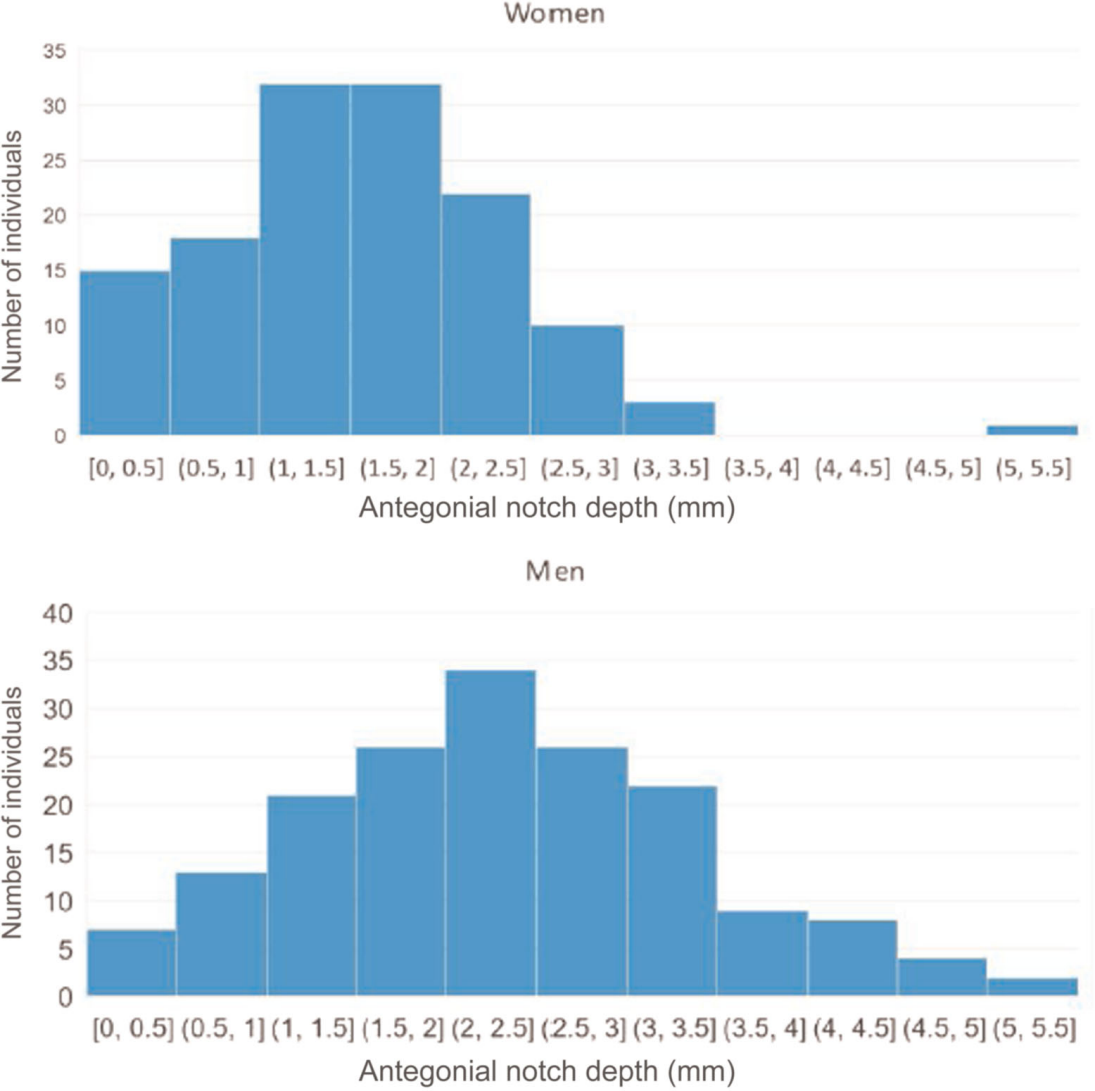
Variation in antegonial notch depth for females and males. The *y*‐axis represents the number of individuals and the *x*‐axis the antegonial notch depth (in mm).

**Figure 4:**
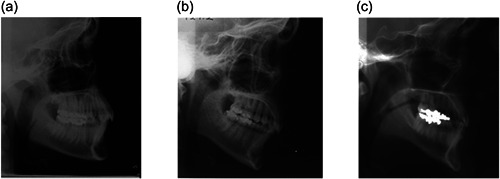
Examples of different antegonial notch depths: (a) shallow, (b) average, and (c) deep.

Among the 302 untreated subjects at 17/18 years, antegonial notch depth ranged from 0 to 5.3 mm (1.97 ± 1.03 mm) (Figure 3). Examples of different notch depths are shown in Figure 4.

There was a significant difference between males and females with regard to notch depth. The antegonial notch was deeper in males (2.3 ± 1.1 mm) than in females (1.5 ± 0.7 mm) (*p* < .001).

**Figure 5:**
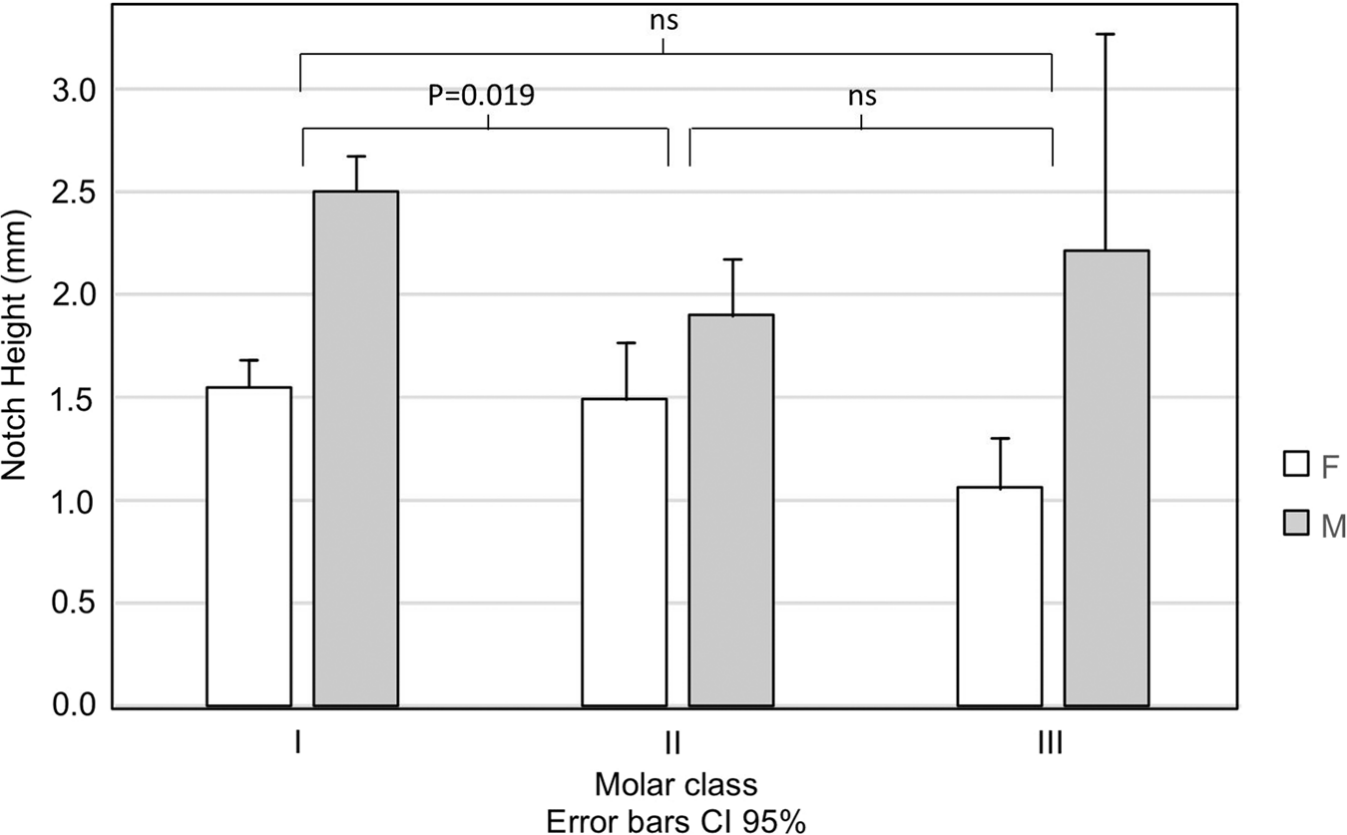
Antegonial notch depth in males and females according to Angle Class for the 302 postadolescent subjects. Error bars represent 95% confidence intervals. F, female; M, male; ns, not significant.

When dividing the individuals into Angle Class I, Class II, and Class II malocclusion, no significant differences were found in antegonial notch depth between Angle Class III and the other classes. There was a significant difference between Angle Class I and II, with notch depth being on average 0.33 mm deeper in Class I individuals (*p* = .019) (Figure 5). No significant difference between Angle Class I and Class II was found however when looking only at the female subsample.

### 3.3 Longitudinal deep antegonial notch changes

**Figure 6:**
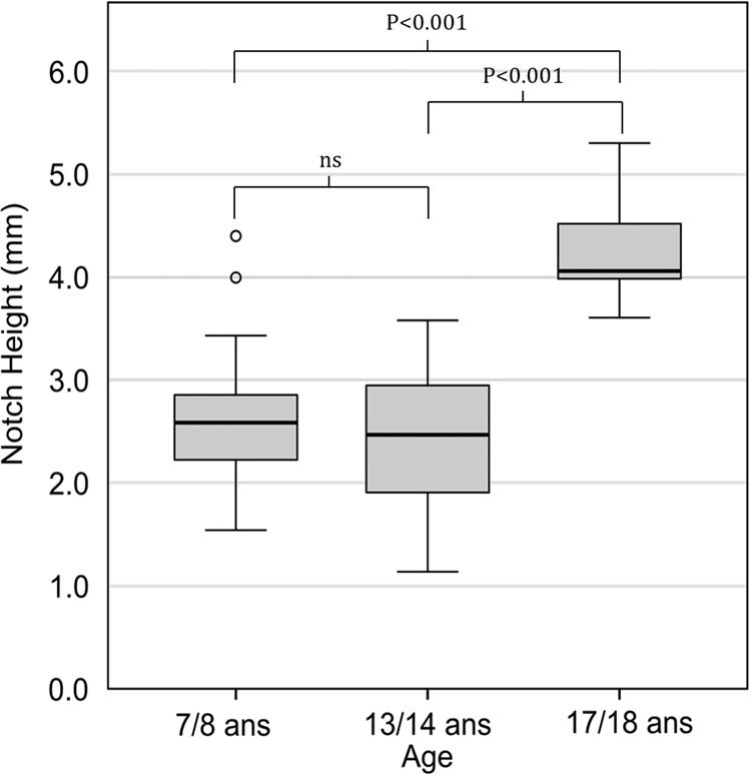
Box and whisker plots showing the magnitude of deep antegonial notches, with notch depths shown at the ages of 7/8, 13/14, and 17/18 years. ns, not significant.

The threshold for deep antegonial notches was calculated to be ≥3.6 mm, which corresponded to depths >1.5 standard deviation above the mean. The individuals with the deepest antegonial notch all happened to be male. There were 21 males in total with deep notches. Nineteen of these 21 males had Angle Class I sagittal relationships, and the remaining two had Angle Class II. While no statistically significant differences were found between the ages of 7/8 and 13/14 in antegonial notch depth, there were significant differences between the ages of 7/8 and 17/18 (*p* < .001) and between the ages of 13/14 and 17/18 (*p* < .001) (Figure 6).

With regard to notch typology, 15 out of the 21 subjects at 17/18 years showed Type 1, while 6 showed Type 2, and none showed Type 3 morphology. Looking at the longitudinal changes of notch morphology, the analysis showed that this did not significantly change between the ages of 7/8 and 17/18, with the vast majority of notches (86%) maintaining their initial typology.

No significant correlations were found between the antegonial notch depth of the 21 males with deep notches and sagittal and vertical skeletal relationships.

## 4 DISCUSSION

This study provides normative data for antegonial notch depth in an orthodontically untreated postpubertal population (17/18‐year‐olds). It can be observed that males have a deeper antegonial notch on average than females. This notch depth seems to increase in magnitude principally during or after the adolescent growth spurt, between the ages of 13/14 and 17/18, whereas prepubertally (between the ages of 7/8 and 13/14) there is little increase in depth. The morphology of the notch however seems to remain relatively constant throughout growth.

The deepest antegonial notches, greater than 3.6 mm, were only found in male subjects. This may lead to the hypothesis that the increase in antegonial notch depth may be linked to the increase in the masticatory muscle mass, observed principally in boys during puberty (Kiliaridis et al., 1993; Raadsheer et al., 1996). The presence of deeper antegonial notches in male individuals has also been found by Osato et al. (2012), who interestingly found that this was associated with stronger masticatory force. Another possibility may simply be that males tend to have bigger mandibles and thus deeper antegonial notches.

Deep antegonial notches seem to deepen during the pubertal growth period, according to the present results. Singh et al. (2015) found that in individuals from 10 to 19 years of age, there was no significant correlation between age and notch depth. Their results may simply be as such because the deepening occurs at a specific time period in growth and does not increase linearly. In the present study, no significant changes in notch depth occur between the ages of 7/8 and 13/14 in the deep notch sample, but only after the ages of 13/14.

This lack of change from 7/8 to 13/14 years in the present deep antegonial notch sample may indicate that there is no or little correlation between general craniofacial growth and the deepening of the antegonial notch. This is corroborated by the lack of correlations between antegonial notch depth and vertical or sagittal skeletal characteristics. In their longitudinal study, Kolodziej et al. (2002) concluded that antegonial notch depth is not an adequate indicator for predicting future craniofacial growth potential.

On the other hand, some authors have found that antegonial notch depth is related to craniofacial morphology. Davidovitch et al. (2016) looked at the antegonial notch in postpubertal subjects and found that those with hyperdivergent skeletal patterns had deeper antegonial notches, while those with hypodivergent patterns had shallower notches. Mangla et al. (2011) found similar differences in their total sample, but when looking solely at the male sample, found no significant differences in antegonial notch depth between hyperdivergent and hypodivergent individuals.

The deepening of the antegonial notch may, therefore, be a phenomenon of bone apposition at the gonial angle, which is the site of the muscular pterygomasseteric sling attachment, or bone apposition beneath the mandibular symphysis and the anterior third of the mandibular body, which accentuates the prenotch curvature. The mass and orientation of the masseter, medial pterygoid, and perhaps even the temporalis muscles may partly dictate the size and shape of the gonial angle (van Spronsen et al., 1997), which in turn can influence the development of the antegonial notch. This result supports the hypothesis that the increase in masticatory muscle mass during puberty, which is genetically larger in males, may lead to the development of deep antegonial notches (Kiliaridis et al., 1993; Raadsheer et al., 1996).

There is some evidence stating that following condylar bone changes and shortening of the ascending ramus, a deepening of the antegonial notch occurs (Yamada et al., 2005). One possible hypothesis explaining this deepening of the notch is that the pterygomasseteric sling maintains its initial proportions, inducing bony apposition at the level of the gonial angle while condylar changes occur, thus deepening the antegonial notch. This goes along with the notion that muscular stimulation plays an important role in antegonial notch development.

With regard to antegonial notch typology, Porwolik et al. (2015) mentioned in their study that the most frequent type in males and females was Type 3, while Type 1 was the least frequent. In our study, however, looking at those with the deepest antegonial notch, the majority showed Type 1, while no subjects showed Type 3 morphology. It would be interesting to investigate if there is a link between deep antegonial notches and their typology, and this remains to be investigated in a larger sample.

The development of deep antegonial notches in healthy individuals with normal growth may differ from that seen in individuals with pathological craniofacial growth, leading to the development of deep antegonial notches. It is also not known if there is a difference in antegonial notch typology when comparing these two groups of individuals. Singh et al. (2011) mention that deep antegonial notches in patients with TMJ ankylosis may be the result of the mandible remaining in a backward position, which causes deposition of bone at the gonial angle and resorption in the anterior region of the mandible. Whether this phenomenon of resorption also occurs in healthy populations is not clear.

The limitations of the current study include its retrospective nature and the use of historical subjects from growth studies carried out several decades earlier. One other limitation is the quality of the cephalometric radiographs obtained from these growth studies, which was not always optimal. However, the error of the method results showed an insignificant error and so this limitation can perhaps be overlooked. The difference seen in antegonial notch depth between individuals with Class II and those with Class I malocclusion, although significant, needs to be interpreted with caution since the difference was in the order of 0.33 mm, while the error of the method was 0.3 mm. The rather small sample size of the deep antegonial notch group may be another factor limiting the interpretation of the results. A better‐powered study would perhaps show more meaningful results.

Research on the antegonial notch is at present very limited, and there might well be many other factors influencing its depth and morphology. The potential influence of orthodontic treatment, for example, with functional appliances, or the influence of masticatory function on the antegonial notch is currently not known and would be questions worth investigating. Equally, the potential influence of the antegonial notch with treatment outcomes is also largely unknown.

Interestingly, it has been found that increased activity of the masticatory muscles is correlated with deep mandibular antegonial notches (Tomer & Kishnani, 2011), which may provide some insight into the use of this anatomical characteristic of the mandible as a predictor of treatment outcome such as with the use of functional appliances. Putting our findings into this context may add some information related to the different muscle activity between males and females and differences in antegonial notch depth. It has also been reported that antegonial notch depth may be a possible predictor of mandibular growth (Lambrechts et al., 1996; Singer et al., 1987). Moreover, some data suggest that individuals exhibiting deep antegonial notches may require longer and more comprehensive orthodontic treatment than those with shallow notches (Singer et al., 1987). It would of course be premature at this stage to make any general assumptions in this regard.

As this characteristic anatomical morphological aspect of the mandible has received little interest, at least in research, there is vast scope for further research looking into many aspects of the antegonial notch. It is not known, for example, whether those with deep notches early on in childhood maintain deep notches into adulthood or whether notch flattening occurs. Likewise, the antegonial notch in different ethnic populations has also not been studied, as well as its similarities in families related to a possible genetic determination.

This study did not directly investigate changes in antegonial notching over time in relation to the mandibular growth pattern. The quality of the lateral cephalograms available was, unfortunately, insufficient to allow for structural superimpositions. This would, however, be an important question for future investigation.

## 5 CONCLUSION

Antegonial notch depth shows important variation in postpubertal individuals, with males having deeper notches on average than females. In those with deep antegonial notches, notch depth seems to increase not during prepubertal growth, but during the pubertal growth spurt. We can hypothesize that its increasing depth correlates with the increased muscle mass occurring in males during puberty and the associated increase in bone apposition at the gonial angle process during this period.

## AUTHOR CONTRIBUTIONS

All authors have participated in the conception and design or analysis and interpretation of the data; drafting of the manuscript or revising it critically; approval of the final version of the manuscript.

## CONFLICTS OF INTEREST

The authors declare no conflicts of interest.

## Data Availability

The data that support the findings of this study are available from the corresponding author upon reasonable request.
